# Microencapsulation of Erlotinib and Nanomagnetite Supported in Chitosan as Potential Oncologic Carrier

**DOI:** 10.3390/polym13081244

**Published:** 2021-04-12

**Authors:** Galo Cárdenas-Triviño, Sebastián Monsalve-Rozas, Luis Vergara-González

**Affiliations:** 1 DIMAD, School of Chemical Engineering, Faculty of Engineering, University of Bío-Bío, 1201 I. Collao Ave, Concepción 4081112, Chile; 2 Department of Biological and Chemistry Sciences, Faculty of Medicine and Science, San Sebastian University, Las Tres Pascualas Campus, Concepción 4030035, Chile; smonsalverozas@gmail.com (S.M.-R.); luis.vergara@uss.cl (L.V.-G.)

**Keywords:** chitosan, nanocomposites, drug delivery, biomedical applications, microencapsulation

## Abstract

Microcapsules (MC) based on chitosan (CH) and including nano-magnetite and erlotinib were synthesized. The microparticles were characterized by SEM, FT-IR and TGA. The percentage of encapsulation was determined, as well as its microbiological activity. Finally, the effectiveness of the formulation was evaluated in terms of cell viability and/or toxicity when compared with the reference drug. The formulation used to prepare the microcapsules showed some bacteriostatic properties. The characterization of microcapsules exhibited amorphous spherical shape and average size of 1.29, 1.58 and 1.62 mm for chitosan, chitosan + nanomagnetite and chitosan + nanomagnetite + erlotinib, respectively. The infrared spectra showed characteristic bands of the erlotinib and magnetite, confirming its internalization. The thermogravimetric analyzes indicated that the materials do not undergo changes at optimum working temperatures. The HPLC analysis showed a 52% of encapsulation. Finally, the formulation probed had lower effectiveness and less cytotoxicity, than the drug without encapsulating “in vitro” studies. For that reason several assays are in progress.

## 1. Introduction

In relation to drug treatment, the type of carcinoma will influence on choice of both first-line pharmacological treatment as well as type of maintenance. In the context of chemotherapy, erlotinib is used in treatment of metastatic non-microcytic lung cancer. Although its use is not front-line, it has shown favorable results such as maintenance or support therapy [1].

Erlotinib is an antineoplastic drug which has a mechanism of action that specifically inhibit tyrosinkinase, an enzyme presence in the epidermal growth factor receptor (EGFR). These enzymes are involved in the processes of proliferation and cell death. It is said, the deregulation of this activity is involved in cancer pathogenicity, favoring a excessive proliferation [2]. This drug prevent the deregulated activity of proteinkines to avoid perpetuation from the beginning. Erlotinib has been approved by the Food and Drug Administration (FDA) as a drug for maintenance treatment after platinum chemotherapy in patients with non-microcytic lung cancer. Clinical studies support the efficacy of the drug as maintenance therapy, improving disease progression-free survival. However, it has toxic effects.

Recently nano and microparticulate systems have aroused great interest from the scientific world, with important and potential applications in the field of medicine. The microcapsules are defined as a dispersion particle or solid particles with a size in the range of 1–1000 µm [3]. Furthermore, Da Silva et al. (2014) [4], establish that capsules can be classified according to their size: macrocapsules (>5000 µm) and microcapsules (0.2–5000 µm). From microparticles it is possible to differentiate between microspheres and microcapsules; the first refers to spherically empty particles while microcapsules are particles which have coating material surrounds a nucleus [5]. Microparticulate systems are designed to mimic cellular behavior in terms of mechanics, topography and cellular morphology; even having the feasibility of modifying the particle surface for various purposes, including regenerative medicine, drug diagnosis and delivery systems [6].

In drugs transportation, both microencapsulation and nanoencapsulation, it allows to provide protection against the environment, stabilization and even evade elimination processes; increasing the bioavailability of drug. These formulations form can deliver the drug to specific sites or organs, where small quantities of the drug would be needed and, as a result, have a decrease in adverse effects having an undoubtedly positive impact on drug therapy. The basis of these systems are materials with certain requirements of biocompatibility and biodegradability. Biodegradable materials ideally remain in the body for as long as they perform their function and then disappear without intervention [7]. Size and morphology of these systems play an important role. As set by Beltran (2017) [8] a spherical and regular shape allows to control and predict release, due to a small contact surface. In the opposite way, an irregular shape will have a large surface area, so the release will be faster. Then size is directly related to the amount of drug they transport.

Both nanoparticles and microparticles are prone to certain disadvantages. Such disadvantages fall to differences in absorption, removal, membrane passage and even toxicity. In order to improve their properties it has been developed the Trojan systems, which can be understood as smaller particles suspended or supported in a larger particle, defined by Anton, et al. (2012) [9].

Chitosan is a biopolymer soluble in aqueous medium with weak acid, due to protonation of its amino groups that make it in a cationic polyelectrolyte [10]. Is a substance that lacks toxicity and its molecular weight emerges as a very important feature because both solubility and viscosity depend on it [11]. In encapsulation of drugs, the great advantage of using this polymer is the possibility to modify the surface of the resulting particle.

Parsian et al. (2016) [12] in one of their tests showed that release of the drug gemcitabine from chitosan-based nanoparticles was significantly high at pH 4.5 (65.4%) compared to 5.2 pH solution 33% was observed. This is favorable, especially in solid tumors, as cancer cells have an unusual metabolization of glucose, producing lactic acid, which is transported out of cell within the extracellular fluid of the tumor microenvironment, acidifying this medium [13].

Magnetite is an iron ore, belonging to group of oxides and possess magnetic properties. Certain magnetic nanoparticles present so-called super paramagnetic properties, having magnetic behavior only in the presence of an external magnetic field. This property has been observed in particles smaller than 15 nm, as nanoparticles of iron oxide, which are called by the scientific world as super paramagnetic iron oxide nanoparticles (SPIONs). Other authors have established that super paramagnetism behavior is observed in particles with size smaller than 30 nm [14]. This feature is very important in biomedical applications, since the permanent magnetic behavior of nanoparticles within the body, could be destructive when the magnetic field is removed.

SPIONs are the only FDA-approved nanoparticles for clinical use, which include a wide range of biomedical applications [15] and they are used as contrast agents in magnetic resonance imaging [16]. In a contrast medium, nanoparticles which carry the drug, can be subject to spatial manipulation due to an external magnetic field, achieving to concentrate the bioactive molecule in a target area or organ, making therapy more selective and specific.

The great disadvantage of conjugating drugs to magnetic particles is related to their large surface-to-volume ratio, and its high surface energy, tending to get agglomerated, which may interfere in the natural course of the bloodstream. In addition, they will find the sequence directly with the biological environment, being able to have certain repercussions in terms of cytotoxicity and genotoxicity [15]. To control these situations and for clinical applications to be effectively achieved SPIONs are coated with a material that allows adequate biodistribution and ensures biocompatibility.

However, it is necessary to identify the potential cellular damage involved during its use, both in the short and long term. Though, the iron (Fe) is an element present in the human body, which is in constant homeostasis thanks to the various metabolic pathways of the same. The great concern arises with the overload of that element, together with the accumulation in a certain tissue. The consequence of the iron accumulation, could cause a homeostasis imbalance, which would trigger the mentioned cellular responses. The doses of iron into the body will vary between 1.25% to 5.0% of total iron reserves in human body. SPIONs are presumed to exhibit minimal toxicity, as they can be recycled and/or discarded by natural biodegradation of the body [16]. In the body, SPIONs will be presumably degraded into iron ions by the action of lysosomes, where the released iron will be metabolized in the liver and subsequently used in the formation of blood cells or excreted by renal route.

Actually, over 100 types of cancer such as: skin cancer, breast cancer, lung cancer, colon cancer and prostate cancer and many others. NPs can effectively be used in the cancer treatment. The capacity of the NPs to get linked to the metals, minerals, and drugs makes it as a novel material to treat cancer cells. Cancer is nowadays one of the most worldwide devastating diseases, and the severe treatment procedures like surgery, radiation, and chemotherapeutic drugs often destroy healthy cells and produce toxicity to humans [17]. The NPs absorbs light and convert into heat which is later on applied to orient and destroy specific cancerous cells.

Saratale et al. [18] reported a green and easy methodology to obtain silver nanoparticles (AgNPs) at room temperature taking the aqueous extract of orange citrus x clementine (OPE). Furthermore, the obtention of AgNPs was carried out by optimization of experimental parameters and characterization of particle size mostly circular shape by HRTEM and between 15–20 nm. The antibacterial activity against several bacteria (*Escherichia coli, Bacillus cereus and Staphylococcus aureus*) and antioxidant properties of these silver nanoparticles were studied. There are several natural products with the capability to control the cancer proliferation and could be alternative for cancer treatment without other secondary problems.

The OPE-AgNPs were stable for six months. In vitro experiments exhibited good biocompatibility and open the possible for biomedical research. Several studies showed that OPE-AgNPs possess cytotoxicity against tumor rat C6 cells with 50% of mortality at 60 μg/mL. These results will open several opportunities in biomedical fields.

Since there are many intra hospital infections we tested the most abundant bacteria in our systems. The aim of this work was to evaluate the behavior of passive drug release such as erlotinib from magnetic microcapsules to evaluate the possibility of applications for cancer chemotherapy treatment. Next step will be the application of magnetic field to evaluate the behavior of the drug with cancer lung cells.

## 2. Materials and Methods

### 2.1. Microcapsules Synthesis (Ionic Gelation Method)

The preparation of the microcapsules was performed using nanomagnetite (97%, Merck), chitosan, potassium hydroxide flakes, acetic acid and erlotinib hydrochloride (98%, Sigma Aldrich, St. Louis, MO 63178, USA, using ion gelation method [19]. These 14 mg of Erlotinib were dissolved in 10 mL of water (with drops of heteric acid). Then 100 mL of prepared chitosan solution (CH 4%) was added to the mixture and finally added 0.1026 g of nanomagnetite. The mixture was stirred for 3 h at 500 rpm with a mechanical stirred. The final volume obtained in 110 mL (10 mL of water with the drug + 100 mL of CH 4%) so the concentration of drug in the mixture was 14mg/110mL, the mixture which the capsules were precipitated in the KOH 0.5M solution. Three mixtures are prepared: the first corresponds to chitosan microcapsules, the second corresponds to the target (CH + nanomagnetite) and the third to the mixture (CH + nanomagnetite + drug), which will be called “microparticles formulation”. The concentrations of each component are shown in Table 1.

The initial amount of 14.1 mg of erlotinib encapsulated (corresponding to 9.33% of the maximum does used for lung cancer, and that is 1.28 × 10^−4^% in the microcapsules) was used.

### 2.2. Scanning Electron Microscopy (SEM)

The SEM microscope used was a Hitachi Model SU 3000 equipment (Hitachi, Tokyo, 100-8220 Japan) that provides an X-ray detector. X-ray energy dispersive (EDX) analysis also provides quantitative sample information [20]. The micrographs obtained provide fundamental information, such as size. The size of the microcapsules can be evaluated.

### 2.3. Infrared Spectroscopy with Fourier Transform (FT-IR)

To establish whether the drug and/or components were incorporated into the microcapsules, infrared spectrometry was made with a Perkin-Elmer Spectrum Two FTIR-ATR equipment (Waltham, Massachusetts, USA). The spectra were obtained using diffuse reflectance techniques of erlotinib, chitosan, nanomagnetite and microcapsules of the formulation are recorded.

### 2.4. Thermogravimetric Analysis (TGA)

To determine the physical and chemical characteristics of the microcapsules, a thermogravimetric analysis was performed using a Perkin-Elmer STA 8000 equipment (Waltham, Massachusetts, USA). The weight samples were from 2 to 5 mg which was maintained in a flow of N_2 (g)_ of 50 mL/min and a heating rate of 10 °C/min.

### 2.5. Release of the Dru

The drug to have its effect, it must be released from its carrier. To know how much of the active compound exists available in a system it is necessary to quantify it. The information obtained will have a close relationship with the encapsulation capacity (or efficiency) of the methodology.

#### 2.5.1. Drug Assessment

The methodology provided by Kalyana & Gowri (2011) [21] is used to quantify the drug with a HPLC equipment (Agilent, Infinity 1200, Santa Clara, CA 95051, USA. It is performed to external pattern quantization. The mobile phase consisting of 650 parts of phosphate buffer, 210 parts acetonitrile and 140 parts methanol is prepared. Two standard solutions were prepared whose concentrations were 500 μg/mL. The following concentrations were prepared from the standard: 2, 5, 7, 10 and 20 μg/mL. The duplicate curve was performed.

Sample treatment: the sample was dispersed in a mortar, approximately 100 mg of powder was weighed. They were poured to a 25 mL flask and a volume of methanol is added to carry out its extraction. The flask was arranged in a shaker at 250 rpm for 3 h. Once the extraction is complete, an aliquot is taken, filtered with a filter of 0.22 μL injected. The above procedure was performed in triplicate.

Chromatographic conditions were as follows: Detector: Photodiode; Wavelength: 253 nm. Flow: 1.2 mL/min and Time: 11 min.

#### 2.5.2. Encapsulation Capacity

The encapsulation capacity was obtained using the encapsulation data in the following equation:

EC (%) = ((Quantity encapsulated drug)/ (Final weight of microcapsules))·100(1)

### 2.6. Acute Toxicity in Worms

Simple toxicity tests will give an indication or serve as screening tests to indicate substances that turn out to be toxic to earthworms (*Eisenia foetida*). In the toxicity test, adult worms with well-formed clithelium of the gender *Eisenia foetida* were used. Ten replicates were made per mix, leaving the latter as control. The rehearsal mixes were:

1. Chitosan 4% (in water)
2. Chitosan 4% + nanomagnetite 2% (in water)
3. Chitosan 4% + nanomagnetite 2% + erlotinib 90 µg/mL (in water)
4. Nanomagnetite 0.090% (in water)
5. Nanomagnetite 0.045% (in water)
6. Nanomagnetite 0.0225% (in water)

The filter paper method was used, where 11 glass tubes were taken inside it contained a piece of filter paper. The filter paper contained in the tubes with the above mixtures was soaked with 1 mL and a worm was added. In the eleventh tube the filter paper was soaked with 1 mL of distilled water, acting as a control. They were left in total darkness for 48 h.

### 2.7. Microbiological Assays

The minimum inhibitory concentration (MIC) and the minimum bactericidal concentration (MBC) of formulations against *Staphylococcus aureus*, ATCC 25923; *Pseudomonas aeruginosa*, ATCC 27853; *Staphylococcus epidermidis* ATCC 12,228 and *Escherichia coli*, ATCC 25,922 were determined. The protocols can be seen in Appendix A.

#### 2.7.1. Minimum Inhibitory Concentration (MIC)

MIC determination was made by dilution in broth. The MIC shall correspond to the minimum concentration of the antimicrobial where turbidity is not observed (which implies zero bacteria development) (Taroco, et al. 2006) [22].

#### 2.7.2. Minimum Bactericidal Concentration (MBC)

“MBC is defined as the minimum concentration of an antibiotic that, in a predetermined period of time, is capable of inducing the in vitro death of 99.9% of a bacterial population” [22], and was performed on samples that showed MIC. The mixtures to be evaluated were:

A: Chitosan 4% + nanomagnetite 2% + erlotinib 90 µg/mL.
B: Chitosan 4% + nanomagnetite 2%.
C: Chitosan 4%.

### 2.8. Cellular Viability

The purpose of creating formulations based on antineoplastic drugs is to cause cell death. Thus, to evaluate the effectiveness of the formulation, in terms of cytotoxicity, an in vitro cell feasibility study was performed to compare the effectiveness with the encapsulated drug, using cell lines of lung cancer. The methodology of reduction of bromide from 3-(4, 5-dimethylthiazole-2-ilo)-2, 5-di phenyltetrazol (MTT), where is evident a color changed (blue/violet) is mediated by the metabolic activity of the cells [23]. A mass concentration of macrocapsules generated by milliliter was used for feasibility testing. A concentration suspension of 8 mg/mL was prepared using dimethylsulfoxide (DMSO) as a solvent, to subsequently dilute and achieve concentrations of 40 μg/mL, this being the first concentration to be evaluated. In a gutter, dilutions were performed on base 2 to be incorporated into the wells and carry out the test. You can see more details in Appendix A. All the statistical treatment to this trial has been carried out and the results will also be observed in tables in Appendix B.

## 3. Results

### 3.1. Scanning Electron Microscopy (SEM)

**Figure 1:**
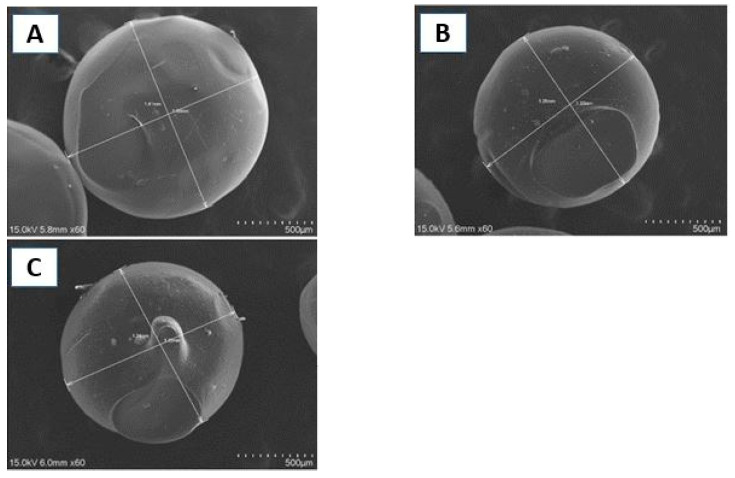
SEM micrographs. Chitosan microcapsules (**A**); Chitosan microcapsules + nanomagnetite (**B**) and Chitosan microcapsules + nanomagnetite + drug (**C**).

The average diameter of the front plane of each observed particle in the Figure 1, calculated as well as the volume are showed in Table 2.

### 3.2. Infrared Spectroscopy

To find out whether the components were internalized into the microcapsules, the infrared spectra of the components and formulation were obtained.

Table 3 summarizes the main groups that can be identified; the band at 3250 cm^−^^1^ corresponds to the stretching vibration of the N-H bond of the secondary amine and at 1499 cm^−^^1^ the bending band of the N-H bond of the same is seen. The band at 2104 cm^−^^1^ corresponds to the acetylene group present in the erlotinib molecule. The bands at 1150 cm^−^^1^ and 1033 cm^−^^1^ correspond to the vibration of an ether alkyl link. The band at 1129 cm^−^^1^ would correspond to the stretching vibration of the C-O bond in the dialkyl ether present in the molecule.

In Table 3 the bands of chitosan at 3144 cm^−^^1^ correspond to the stretch of the O-H bond. At 2872 cm^−^^1^ a band is observed that would correspond to the stretching of the C-H bond. At 1588 cm^−^^1^ one of the bands belonging to the deformation of the N-H bond is observed. The C-O stretching tension bond yields an absorption band at 1062 cm^−^^1^.

The Fe-O link in the magnetite (Fe_3_O_4_) usually shows a band around 600 cm^−^^1^. In Table 3 it is possible to see a band at 662 cm^−^^1^ [24].

The similarity of the spectra in Table 3 (corresponding to the chitosan spectrum) is remarkable. The main reason is because the component that is in greater proportion is chitosan and assuming a homogeneous distribution, the drug only corresponds to 0.205% of the total mass of microcapsules. A shoulder at 2100 cm^−^^1^ corresponding to the acetylene group or erlotinib.

To assess whether the drug was internalized in the prepared capsules, the spectra were compared. The band at 3250 cm^−^^1^ belongs to the N-H bond tension of the secondary amine of the erlotinib molecule is not appreciated, possibly due to the overlap of the bands generated by the stretching of the O-H bond of the chitosan molecule.

The band at 1499 cm^−^^1^ of the secondary amine bending is also not appreciated by overlapping the N-H link bending band of the primary chitosan amine. The band at 1254 cm^−^^1^ of the alkyl aril ether link is observed as “shoulder”. The second band at 1051 cm^−^^1^ would be overlapped with the band generated by the C-O bond tension of the chitosan molecule. Finally, the absorption signal at 660 cm^−^^1^ corresponding to the Fe-O link of the magnetite.

**Figure A2:**
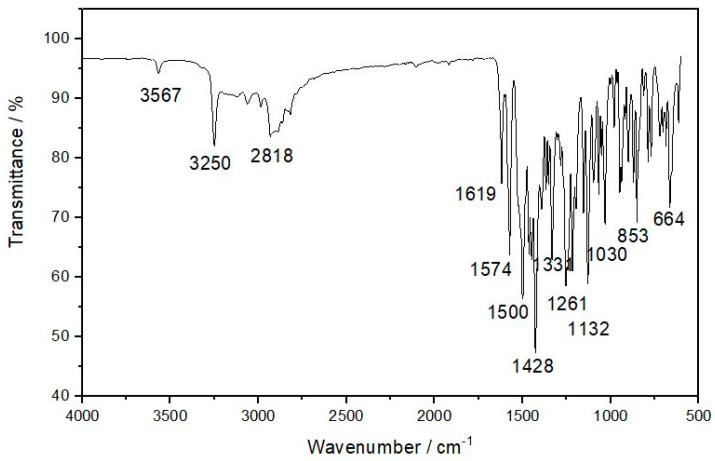
Erlotinib infrared spectrum.

**Figure A3:**
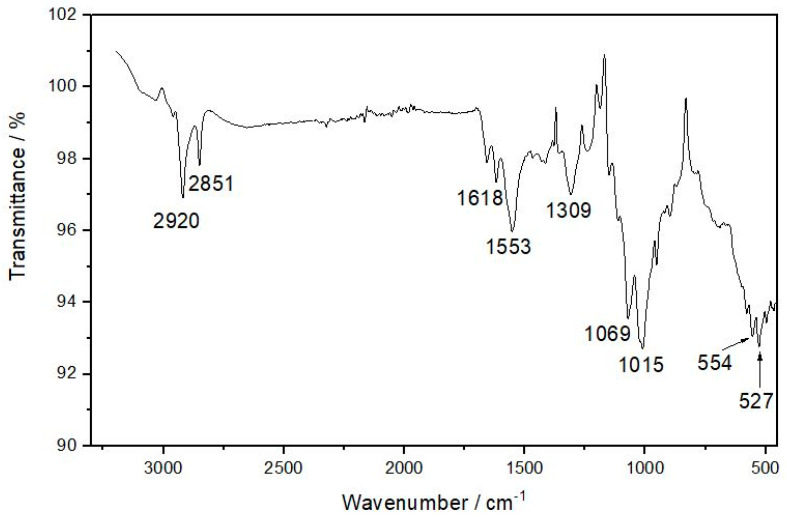
Chitosan infrared spectrum.

**Figure A4:**
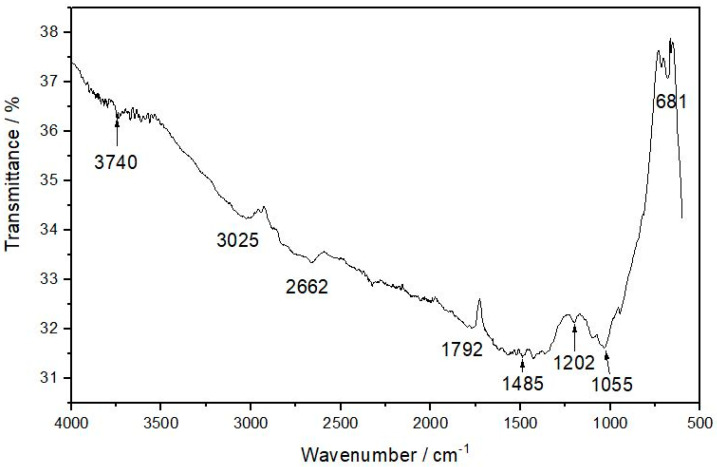
Magnetite infrared spectrum.

**Figure A5:**
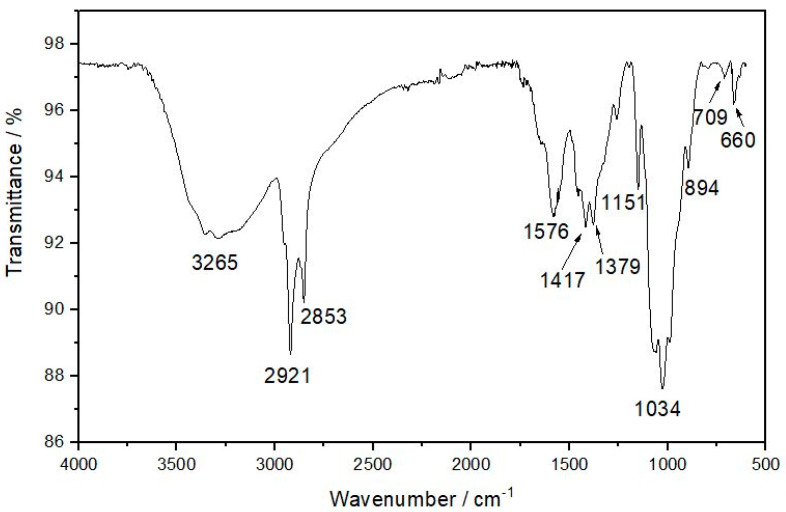
Infrared Spectrum of formulation.

**Figure A6:**
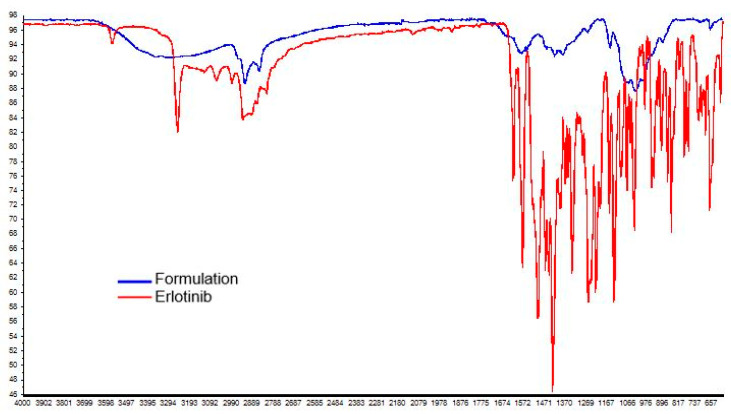
Erlotinib infrared spectra and formulation overlapping.

It is possible that, along with the overlap, the amount of analyte to be evaluated may be conditioning in the signal strength. All spectra are shown in Appendix B Figure A2, Figure A3, Figure A4, Figure A5 and Figure A6.

### 3.3. Thermogravimetric Analysis (TGA)

**Figure 2:**
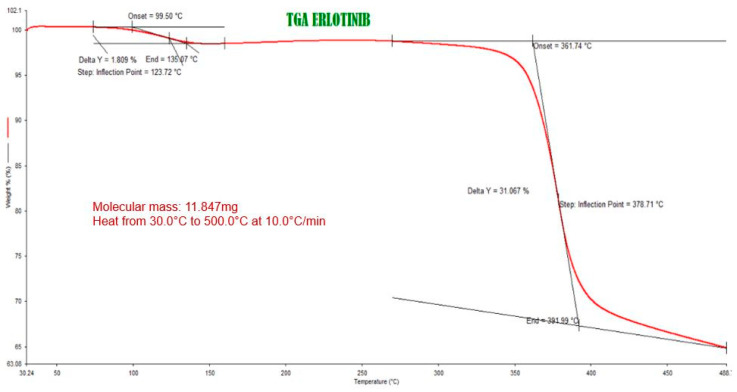
Erlotinib thermogram.

**Figure A7:**
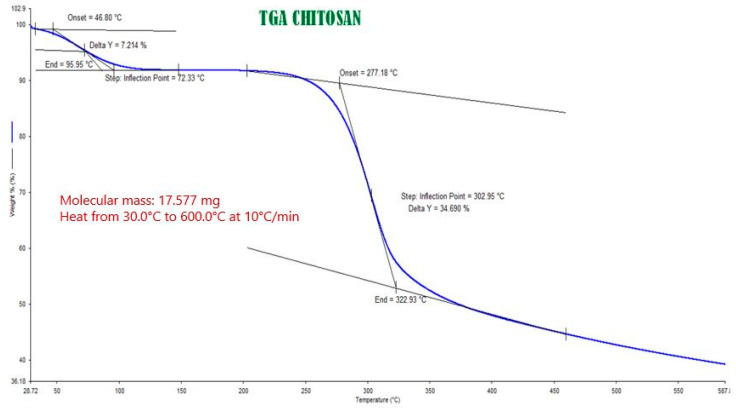
Chitosan Thermogram.

**Figure A8:**
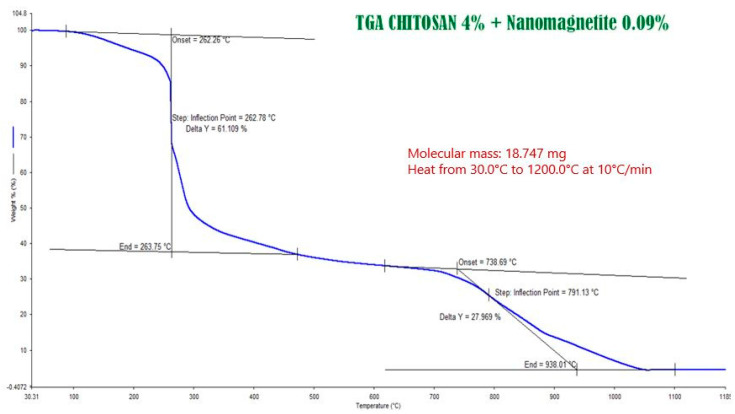
Chitosan 4% + Nanomagnetite 0.09% Thermogram.

**Figure A9:**
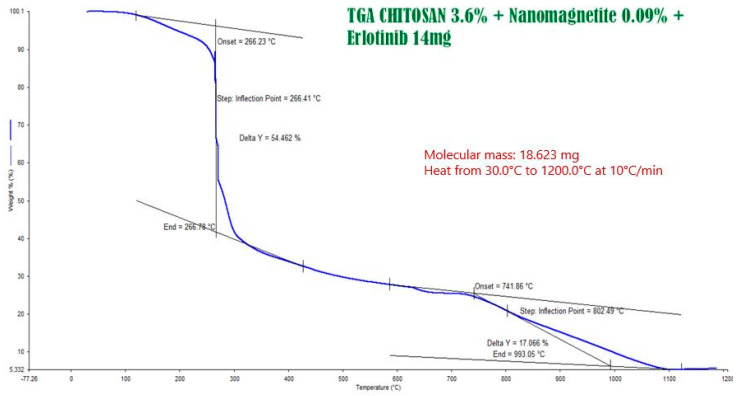
Chitosan 3.6% + Nanomagnetite 0.09% + erlotinib 14mg Thermogram.

Figure 2 presents the thermogram obtained from the drug Erlotinib, using a temperature ramp of 10 °C/min in a range of 30 to 500 °C. Appendix B Figure A7, Figure A8 and Figure A9 show thermograms obtained from chitosan; microcapsules of chitosan + nanomagnetite and microcapsules of chitosan + nanomagnetite + Erlotinib, respectively. In the thermogram in Figure 2 you can see the decrease in the mass of the sample depending on the temperature. Two turning points stand out; the first occurred at 123.72 °C and the second at 378.71 °C with their Y deltas: 1.809% and 31.067%, respectively. Knowing the initial mass can determine mass variation as:

Table 4 summarizes the data obtained from the thermograms.

Looking at Table 4, thermogram 1 and Figure A8 and Figure A9 in Appendix B, it is apparent that around 260 °C there is a considerable loss of mass, due to rings decomposition from erlotinib and then comes a second turning point around 800 °C. Both samples contain nanomagnetite in their formulation, an important fact to consider. Looking at the thermogram in AB.6 there is a clear difference in the decomposition points in the case of chitosan only. The first point arises around 70°, which is humidity, while the main second one occurs at 302 °C.

In the case of the drug, the largest observed change is around 123 °C, then another slope is seen at 378 °C. It should be noted that the thermogravimetric analysis for the drug was only carried out up to 500 °C, so it did not allow to observe the variation of the mass at higher temperatures. Despite this, considering the temperatures of the analyses, it can be inferred that all samples to be evaluated do not undergo significant changes in their masses at normal working temperatures.

### 3.4. Drug Valoration

**Figure 3:**
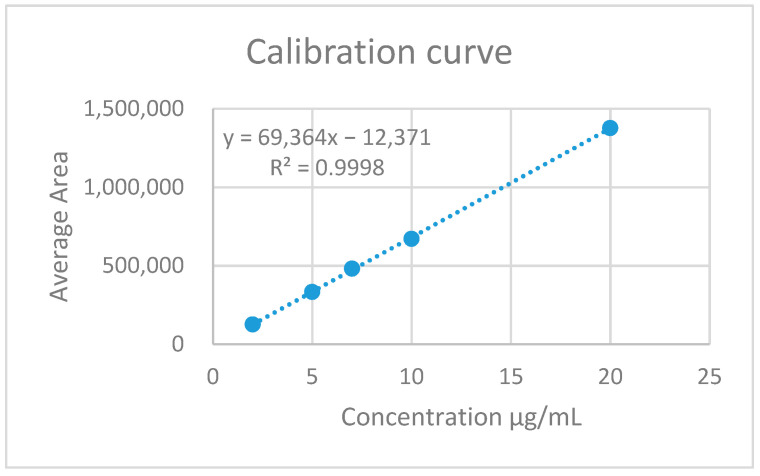
Calibration curve of Erlotinib standard.

In order to determine the amount of drug in the particles, drug was quantified using liquid chromatography, using an external pattern. Standard concentration and areas obtained are presented in Table 5. From the data obtained, a calibration curve was carried out (Figure 3).

From the HPLC areas the amount of drug could be determined (see Table 6). Subsequently, the absolute amount of the drug is calculated. The results are shown in Table 7.

Encapsulation Capability (E.C.):

E.C. (%) = 0.205.

### 3.5. Acute Toxicity

Several studies have used magnetite to generate magnetic particles, in which a concentration of 2% has been used. For the present work it is considered that nanomagnetite that would have a higher surface/volume ratio, making it more reactive, so it is decided and decrease the concentration, even though less than half. In pre-trials, the toxicity of a nanomagnetite mixture at 0 has been evaluated. 25% in water in earthworms of the genus *Eisenia foetida* was used. For each concentration ten test tubes were used with 1 mL of water and a filter paper with the microcapsules powder and nanomagnetite impregnated on it and a warm on each of them. The test tubes were kept at 20 °C for 48 h. The results yielded death in all the assays, which is the reason to evaluate again using a concentration at 0.09%, 0.045% and 0.0225%. All of the worms remained alive in all the concentrations evaluated. Similarly, chitosan, chitosan+ nanomagnetite and chitosan+nanomagnetite+erlotinib were tested exhibiting no dead under this experimental conditions.

### 3.6. Microbiological Assays

The Minimal Inhibitory Concentration (MIC) of mixtures that will give rise to microcapsules, against bacteria strains of *S. aureus*, *P. aeruginosa*, *E. coli* and *S. epidermidis*, was determined, showing no differences between chitosan alone or in mixture with nanomagnetite or drug. The results are shown in Table 8.

Mixtures corresponding to 1, 2 and 3 represent “stock” mixtures of 4%, nanomagnetite 2% and erlotinib 90 µg/mL; 4% chitosan and nanomagnetite 2% and chitosan 4%, respectively. Three replicas were made. It is possible to observe that the concentrations of the components to which inhibition occurs are identical in all mixtures. The MIC is due mainly to chitosan and the additives it does not improve the bactericide properties.

### 3.7. Cellular Viability

**Figure 4:**
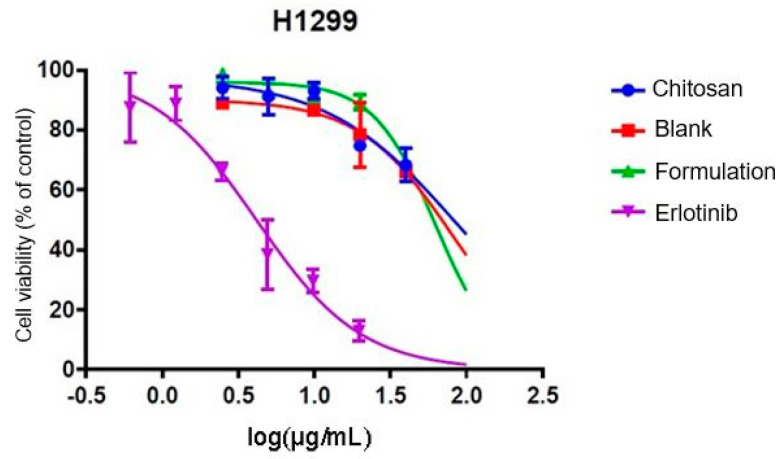
Cell viability against concentration of formulations.

Cell toxicity analyses of synthesized microparticles were performed using the MTT methodology in “eternal” human lung cancer cells of the H1299 line, at the Institute of Pharmacology and Morphophysiology of the Austral University of Chile. Cellular viability (in percentage) versus concentration logarithm is shown in Figure 4.

In Table 9, effective concentration values 50 (EC_50_) are shown.

For the analysis of data, it was chosen to evaluate only the doses of 20, 10 and 2.5 μg/mL, since the results of the doses of 5 and 1.25 μg/mL contain data that do not follow the trend and are considered “outlayers”.

Table 10 shows the results of cellular viability, as a percentage with respect to a control, caused by chitosan microcapsules, blank microcapsules (chitosan + nanomagnetite), microcapsules of the formulation (chitosan + nanomagnetite + erlotinib) and finally the encapsulated drug. The concentrations evaluated correspond to microcapsule weight per volume unit.

In order to assess whether the difference between formulations, according to their absorbance, is statistically significant the ANOVA statistical model of a factor was applied. Statistical differences are determined according to the dose as summarized in Table A1 in the Appendix B.

To get the ANOVA, Table A2 in the Appendix B summarizes the values. Using the “Real Stats” tool of the Excel software, the data corresponding to the ANOVA table was obtained, from which the *p* value was obtained.

As can be seen in Table A2 the value of probability *p* is less than the value of s in all the concentrations evaluated, indicating that there is a statistically significant difference, so that the null hypothesis is rejected, resulting in acceptance of the alternative hypothesis.

In addition, and in order to assess whether there is significant variation between the different formulations, multiple comparisons were made between the groups using the Tukey test, the data obtained are shown in Table 11.

It is observed that the value of *p* is less than 0.05 when comparing the groups: Chitosan-Erlotinib; Blank-Erlotinib and Formulation-Erlotinib at all doses, indicating that there are statistically significant differences. The opposite occurs when comparing the groups: Chitosan-Blank; Chitosan-Formulation and Blank-Formulation, where the *p* value is greater than 0.05.

It is clear to witness that the prepared formulation is no more effective than the reference drug (one-way ANOVA, *p*: 0.05). In Table 9 you can see that the EC_50_ value of the reference drug is approximately 15 times lower than the formulation, which results in the reference drug being 15 times more effective than the formulation. EC_50_ is a parameter that allows to identify the potency of drugs when faced in a dose-response graph. This term refers to the amount (dose) of the drug needed to cause the effect on 50% of the population.

In the results of multiple comparison it can be observed that there is no significant difference between the absorbances of: chitosan, blank (chitosan + nanomagnetite) and formulation (chitosan + nanomagnetite + erlotinib). If the absorbance levels are directly related to cellular viability, it would be logical to expect that there would be a significant difference between the chitosan and the formulation and/or blank and formulation, since it is the latter that possesses the active component.

**Figure 5:**
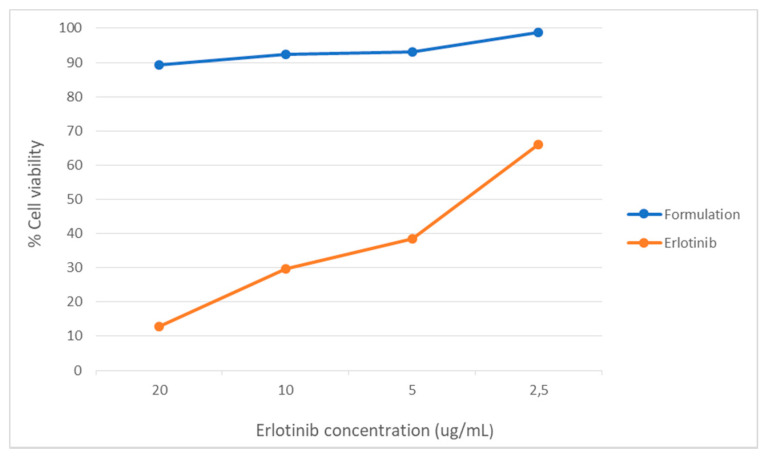
Viability caused by the Formulation and Erlotinib.

For the groups that possess the drug which would be expected to have an impact on cellular viability, the formulation and the reference drug were found. According to the Tukey test when comparing the two groups, a statistically significant difference is seen for all doses evaluated. The graph in Figure 5 shows the difference in cellular viability produced by the formulation and the reference drug according to concentration.

On the other hand, Ahrari, et al. (2010) [25] classifies the toxicity (effectiveness) of formulations based on cellular viability as shown in Table 12.

Table A3 in the Appendix B shows the cytotoxicity of formulations based on the classification of Ahrari, et al. (2010) [25].

## 4. Discussion

The microcapsules exceeded slightly 1000 µm in diameter, a somewhat high value for an intravenous administration, taking into account the risk of embolism. Chandna, et al. (2013) [26], establish that a particle size of less than 4 µm is an adequate size for particles to filter through blood vessels. The drug to cause a desired effect, it needs to be internalized in the cell. In contrast, nanoparticles, due to their size, have the peculiarity of come across membranes more easily than a microparticles. Crossing membranes makes it more feasible to deliver the drug to a site of interest. Derakhshandeh and Fathi (2012) [27] demonstrated that the internalization of gemcitabine by the cell is significantly higher when the drug is nanoencapsulated in a chitosan system. On the other hand, although it cannot be properly observed, the morphology of the particles resembles a disc or a lentil. Jelinek (2015) [16] establishes certain advantages and disadvantages possessed by the different particle shapes when traversing membranes depending on the contact surface of the particle. However, in this case the form does not play a leading role, since the resulting size makes it physically impossible to consume the particles in their full form. The disc shape can be explained due to the concentration of chitosan used in addition to the loss of shape of the drop that comes out in the preparation system.

In relation to particle characterization, the internalization of the drug and the magnetic component was confirmed by infrared (FT-IR) analyses. While the intensity of the peaks is not optimal, when analyzing the spectra in detail and in an overlapping way you can see a band that points to a characteristic group of the molecule of the drug. The low intensity of the bands drug is due to the low mass proportion (0.205%) in the formulation. The band that confirms the internalization of the magnetite is satisfactorily observed in the spectrum of the formulation. The chitosan concentration is shielding the other components bands.

Thermograms show a substantial difference in temperatures at inflection points. Formulations containing nanomagnetite are more thermostable than formulations that do not contain it. In the case of the former, the first observed turning point is approximately 260 °C while the second observed point is around 800 °C. The percentage of mass loss is very similar in both formulations. This could be due to the increased thermal stability of the system by the nanomagnetite microdomains present in the microcapsules. While the chitosan thermogram could indicate greater thermo stability with a first weight loss at 73.22 °C, the loss of mass observed at that point is water. On the other hand, the erlotinib thermogram shows a first decomposition at 123.72 °C, probably to water presence, then at 378.71 °C a second decomposition with a greater loss is observed. The size of microcapsules makes it difficult to meet the therapy objectives, mainly due to the impossibility to penetrate the capsules into the cell (considering that the size of a cell is around 10 μm), so the drug must undergo to a process of release of it. Because of its size, spatial manipulation with an external magnetic field it is also difficult to concentrate the agent on a site of interest, considering the obvious risk of embolism. These are preliminary results, the next step is to prepare the nanocapsules.

The absorbance obtained is proportional to cellular metabolic activity; the cell has increased metabolic activity, resulting in greater viability. It is logic to think that the cell remains less viable compared to formulations containing the drug, since the action of the drug is to cause death and in formulations that do not contain the drug the feasibility rate will be higher. After performing the feasibility test (MTT) and observing the results it can be seen that there is no statistically significant difference between the target and the formulation. On the contrary, there is such a difference between the formulation and the reference drug. There are problems in the release of the active substance or the amount of drug in microcapsules is insufficient, basically due to the methodology used in which the erlotinib is not very soluble in dimethylsulfoxide.

During the test it was not feasible to suspend the microcapsules in the medium, considering the size. The solution to this problem was the crushing and subsequent sifting of the particles (sifting No. 80) to achieve suspending them. It is important to consider, the reference drug was in solution, instead the formulation to be tested was a suspension. As a solution the molecular units of the drug are in unitary and free form, resulting in a higher concentration in the environment. On the contrary, the drug in the formulation was attached to the particles, so mechanisms such as erosion are responsible for releasing it and dissolving it gradually in the middle, with the consequent absorption. By crushing the microcapsules, the matrix of these was completely destroyed, so it would leave mostly exposed to their components. As mentioned, SPIONs tend to clump together, so they should be covered in such a way as to avoid this phenomenon. The concentrations of the formulation that were tested do not represent equimolar amounts of drug, rather represent the concentration of microcapsules. The microcapsules were evaluated as “a whole” and not specifically the amount of drug in them, since, if the transfer concentration of Erlotinib is considered, the formulation would undoubtedly be disadvantaged. Hosseinzadeh et al. (2012) [28] prepared nanoparticles smaller than 200 nm of chitosan/Pluronic^®^ for transportation of the drug gemcitabine, where they observed the prepared system produced a cytotoxic effect on the HT-29 cell line of colon carcinoma, when compared to blank nanocapsules and the isolated drug.

The most effective deliveries are in active release systems along with signaling per action of an external magnetic field [29]. For the purposes of this work only in vitro cytotoxicity was evaluated without application of an external magnetic field. Future studies will assess the effect of the application of a magnetic field on the effectiveness of the formulation.

Although the content of the active substance was quantified, the nanomagnetite content was not determined. Nanomagnetite is the component that will give the magnetic character to the particle, therefore it is indispensable for future testing. Despite this, achieving fairly high values can give rise to toxicity tables. Iron is an essential component in the human body whose metabolic pathways cause proper homeostasis of the human body, but exceeding certain limits can be harmful. That is why the percentage of encapsulation and/or mass balance should be improved and evaluated. By only determining the amount of drug in the formulation the amount of magnetite present is left out. If you get low encapsulation percentages, you will be forced to take more of the formulation to “meet” the dose of the asset, which would involves using large (and unknown) amounts of magnetite.

Knowing the dose of the drug and/or components that is able to inhibit the growth of certain types of bacteria is extremely useful in the research process, especially in which formulations are prepared with possible future applications in patients; patients in which an infectious process may have non-minor consequences [30].

Formulations have been shown to have bacteriostatic activity at the concentrations evaluated. The antimicrobial properties of chitosan are known. The results in Table 8 are striking since the same MIC was found for the four bacterial strains tested. It is known that the antibacterial activity of chitosan depends on factors such as molecular weight and percentage of deacetylation among others. In previous studies we found differences in MICs for the same strains but it was another chitosan (Cardenas, 2020) [31]. Results similar to ours found Jung et al. 2010 [32], who tested 14 strains of Gram positive and negative bacteria, finding very similar or identical MIC values for all strains, which indicates that the antibacterial activity is highly dependent on the characteristics of the chitosan used. According to results our research is oriented to obtain nanocapsules and test their efficiency and orientation in the presence of a magnetic field in contact with lungs cell cancer.

## 5. Conclusions

Infrared spectroscopy, exhibit characteristic bands, corroborating the incorporation of the drug and magnetite in the microcapsule.

The diameters of the microcapsules was increasing according to the amount ofn components. It is also necessary to improve the amount of drug incorporation in the microcapsules to become more effectives.

Even if the encapsulation capacity was not higher, it would undoubtedly have hopeful results when penetrate the cell. In addition, nanoparticles would be ideal candidates for spatial manipulation thanks to an external magnetic field for the same reason.

In future trials, it is necessary to evaluate the cytotoxicity of erlotinib magnetic nanocapsules and how it varies their efficiency when an external magnetic field is applied.

## Figures and Tables

**Table 1:** Component quantities.

Samples	Microcapsules		
	Chitosan	Blank	Formulation
Chitosan	3.6%	3.6%	3.6%
Nanomagnetite	-	0.090%	0.090%
Erlotinib HCl	-	-	14 mg

**Table 2:** Average microcapsules size.

Microcapsules	Average Diameter (mm)	Volume (mm^3^)
Chitosan	1.29 ± 0.01	1.12 ± 0.01
Chitosan + nanomagnetite	1.58 ± 0.01	2.06 ± 0.01
Chitosan + nanomagnetite + drug	1.62 ± 0.01	2.22 ± 0.01

**Table 3:** FTIR bands relevant.

Band	Bond	Frequency (cm^−1^) Chitosan	Frequency (cm^−1^) Nanomagnetite	Frequency (cm^−1^) CH + Nanomag + Erlotinib
1	νOH	3144		3150
2	νC-H	2871		2653
3	νN-H	1588		3265, 1575
4	νC-O	1025		1033
5	νC≡C			2100
6	νC=N			1650
7	νO-CH_3_			2921, 1150
8	νC-O-C			1150, 1033
9	νFe-O		662	660

**Table 4:** Data from thermograms.

Thermo- Gram *	Initial Mass (mg)	Temperature 1st Decomposition Point	Temperature 2nd Decomposition Point	Lost Mass around 1st Point	Lost Mass around 2nd Point
1	11.847	123.72 °C	378.71 °C	0.214 mg	3.680 mg
2	17.577	73.23 °C	302.95 °C	1.269 mg	6.097 mg
3	18.747	262.78 °C	791.13 °C	11.456 mg	5.243 mg
4	18.623	266.41 °C	802.49 °C	10.142 mg	3.178 mg

* 1: Thermogram Erlotinib; 2: Chitosan thermogram; 3: Thermogram microcapsules of chitosan + nanomagnetite; 4: microcapsules of chitosan + nanomagnetite + Erlotinib.

**Table 5:** Peak Areas obtained from the standard.

Standard	[Standard] µg/mL	Area 1	Area 2	Average
A	2	128,839	123,760	126,300
B	5	344,564	322,034	333,299
C	7	511,299	453,135	482,217
D	10	703,748	638,966	671,357
E	20	1,455,979	1,298,030	1,377,005

**Table 6:** Area obtained from the sample.

Sample	Area Average
#1	618,649
#2	617,854
#3	625,811

**Table 7:** Absolute amount of drug.

Sample	Sample Powder (mg)	Drug Concentration (µg/mL)	Drug Quantity in 25 mL(mg)	Drug Quantity in 3576.1 mL(mg)
#1	110.5	9.09	0.22725	7.35
#2	107.9	9.08	0.22700	7.52
#3	111.4	9.20	0.23000	7.24

Total mass spheres: 3.5761 g = 3576.1 mg. Percentage of drug recovered: 52.19%.

**Table 8:** Summary of MIC values for each component and mixtures.

Strains	Mixture *	Chitosan	Nanomagnetite	Drug
*S. aureus*	1	0.0625%	0.0312%	1.40 µg/mL
	2	0.0625%	0.0312%	-
	3	0.0625%	-	-
*P. aeruginosa*	1	0.0625%	0.0312%	1.40 µg/mL
	2	0.0625%	0.0312%	-
	3	0.0625%	-	-
*E. coli*	1	0.0625%	0.0312%	1.40 µg/mL
	2	0.0625%	0.0312%	-
	3	0.0625%	-	-
*S. epidermidis*	1	0.0625%	0.0312%	1.40 µg/mL
	2	0.0625%	0.0312%	-
	3	0.0625%	-	-

* 1 Chitosan + nanomagnetite + drug. * 2 Chitosan + nanomagnetite. * 3 Chitosan.

**Table 9:** EC_50_ values.

	Chitosan µg/mL	Blank µg/mL	Formulation µg/mL	Erlotinib µg/mL
EC_50_	86.44	80.61	63.22	4.184

**Table 10:** Microcapsules cytotoxicity.

	Cellular Viability (Control %)			
Concentration MP (µg/mL)	Chitosan	Blank	Formulation	Erlotinib
1.25	74.77 ± 2.0	91.98 ± 5.3	80.52 ± 4.9	88.84 ± 6.4
2.5	94.04 ± 3.6	89.02 ± 1.3	98.94 ± 3.1	66.20 ± 2.9
5	91.24 ± 6.1	71.63 ± 1.0	93.18 ± 2.4	38.49 ± 11.6
10	93.07 ± 2.7	86.89 ± 1.4	92.44 ± 2.9	29.65 ± 3.8
20	74.82 ± 1.6	78.36 ± 10.0	89.36 ± 2.4	12.94 ± 3.3

**Table 11:** Tukey Test: multiple comparisons.

		Probability Value *p* (α = 0.05)		
Doses (µg/mL)	Groups	Blank	Formulation	Erlotinib
20	Chitosan	0.925370665	0.200942836	0.001574813 *
	Blank	-	0.359918262	0.001270028 *
	Formulation	-	-	0.000692634 *
10	Chitosan	0.275210579	0.995969439	0.00009710 *
	Blank	-	0.338377553	0.000142962 *
	Formulation	-	-	0.00010055 *
2.5	Chitosan	0.41325889	0.428752192	0.002247612 *
	Blank	-	0.084514574	0.004776099 *
	Formulation	-	-	0.001202746 *

* Statistically significant difference.

**Table 12:** Toxicity rating.

Cellular Viability	Toxicity
Greater than 90%	No cytotoxic
Between 60–90%	Light cytotoxicity
Between 30–59%	Moderate cytotoxicity
Smaller that 30%	Severe cytotoxicity

**Figure A1:**
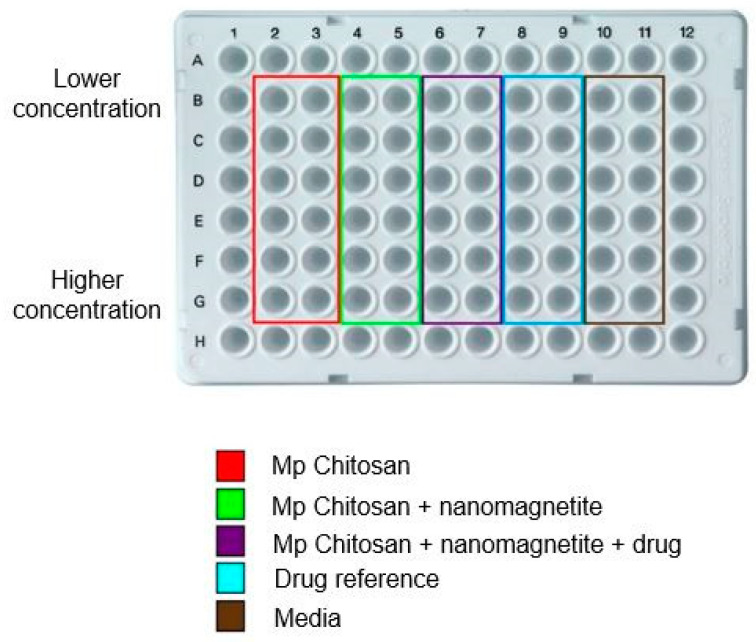
Disposition of composite to test.

## Abbreviations

**MC**	Microcapsules
**CH**	Chitosan
**SEM**	Scanning Electron Microscopy
**FT-IR**	Infrared Spectroscopy with Fourier Transform
**TGA**	Thermogravimetric analysis
**HPLC**	High Performance Liquid Chromatography
**EGFR**	Epidermal Growth Factor Receptor
**FDA**	Food and Drug Administration
**SPIONs**	Super paramagnetic Iron Oxide Nanoparticles
**EDX**	X-Ray Energy Dispersive
**MIC**	Minimum Inhibitory Concentration
**MBC**	Minimum Bactericidal Concentration
**MTT**	3-(4, 5-dimethylthiazole-2-ilo)-2, 5-di phenyltetrazol
**DMSO**	Dimethylsulfoxide

## Appendix A

In this section you will find detailed methodologies for synthesis, microbiological assays and cell viability.

MICROCAPSULES SYNTHESIS METHODOLOGY

The basis of all mixtures corresponds to a 4% chitosan solution in 2% acetic acid.

Preparation of the Chitosan mixture: 100 mL of prepared chitosan solution is measured and 10 mL of acidified water was added.

Preparation of the blank mixture: 100 mL of the prepared chitosan solution is measured 10 mL of acidified water and 0.1026 g of nanomagnetite. The mixture is stirred for 3 h at 500 rpm with a mechanical stirrer.

Preparation of the mixture with the drug: 14 mg erlotinib HCl was dissolved in 10 mL of acidified water then was added 100 mL of the prepared chitosan solution and 0.1026 g of nanomagnetite. The mixture was stirred for 3 h at 500 rpm with a mechanical stirrer.

The drug does not dissolve in the mixture, then a 10 mL of distilled water is used to which drops of glacial acetic acid are aggregated to dissolve the drug. The explanation is based on the drug being a weak base, so acidifying the medium will dissolve. Either the chitosan mixture or the blank mixture, 10 mL more water was added to achieve a level playing field.

Obtaining the microspheres:

All mixtures were kept in constant homogenization with the help of a magnetic bar. 150 mL of a 0.5 M KOH solution is measured in a 250 mL beaker and stirred with a magnetic bar. With a 10 mL syringe a volume of the prepared mixtures is taken. The mixture are dropped into the KOH solution, where the spheres are instantly formed. Once the mixture is exhausted, the resulting spheres are filtered and washed with water. The washing is repeated to obtain a neutral pH. Once the pH was achieved, the microcapsules were filtered, deposited on Petri dishes and brought to dry on the stove at a temperature of 40 °C. This process was repeated for all mixtures.

MICROBIOLOGICAL ASSAYS (MIC and MBC)

Minimal Inhibitory Concentration (MIC) was performed by broth dilution method. Briefly, 11 tubes were used, making 3 replicates for each mixture and for each strain. In the first tube, 950 µL of Mueller-Hinton Broth (M-H) was deposited at twice the original concentration (0.042 g/mL) and 1 mL of the prepared mixtures is homogenized. In the next 10 tubes, 950 L of M-H broth is deposited at the original concentration proposed by the manufacturer (0.021 g/mL). Subsequently 1 mL of the first tube is transfer to the second tube, homogenized and from here again 1 mL is transferred to the second and so on. Therefore in the first tube the concentration of constituent was Chitosan 4%, nanomagnetite 2% and erlotinib 90 ug/mL; and in the last tube number 10 the concentration was 0.0039%, 0.0019% and 0.088 μg/mL respectively. The last tube does not receive antimicrobial solution and serves as a control. The inoculum for each bacteria was prepared adjusting turbidity to standard (McFarland 0.5). 50 μL of inoculum was added to each tube. All samples were incubated at 36 °C between16 and 20 h.

CELL FEASIBILITY ASSAY WITH LUNG CANCER CELLS.

The behavior and cytotoxicity of erlotinib microparticles with chitosan stabilized magnetite are evaluated. Like prepared chitosan microcapsules and white microcapsules.

Immortalized cell cultures of the H1299 line (human cells) are used in RPMI 1640 medium (nourished with fetal bovine serum and antibiotics). The trial was carried out at the Institute of Pharmacology and Morphology of the Universidad Austral de Chile.

Cellular viability was evaluated by reducing the MTT compound.

Preparation of the culture:

The cells of the H1299 line were removed from the original medium with the help of PBS and trypsin to get them off the bottom of the container. Once the take-off is checked (with the help of a microscope) 1 mL of RPMI 1640 medium is added to avoid excess damage by the trypsin. All the contents are deposited in a falcon tube and led to centrifuge for 6 min at 800 G.

After centrifugation the medium is removed and 5 mL of medium is added to resuspend the cells and proceed to count them with the help of a Neubauer camera under the microscope. 2500 cells are added per well.

Loading the samples to be tested:

Prepared microparticles were pulverized and sifted to a size of 180 mm. A concentration suspension of 8 mg/mL is prepared, using dimethylsulfoxide (DMSO) as a solvent, to subsequently dilute and achieve concentrations of 40 g/mL, this being the first concentration to be evaluated. In a gutter, dilutions are made on base 2 to be incorporated into the wells according to the arrangement presented in Figure 2. The test is done in duplicate.

MTT Load:

After 72 h, MTT is added to a concentration of 0.5 mg/mL and incubated for 4 h at 37 °C. Then 100 L of an SDS mixture is added to 10% + HCl 0.01 M to be able to solubilized the formed shaped crystals and appreciate the color. He let himself incubate all night. The next day the absorbances were read at 570 nm.

## Appendix B

In this section the respective figures cited within the text are shown. Besides tables of statistical treatment are here.

**Table A1:** Absorbances to be evaluated.

	Absorbances			
Doses	Chitosan	Blank	Formulation	Erlotinib
2.5 µg/mL	0.847	0.772	0.887	0.598
	0.802	0.789	0.848	0.562
10 µg/mL	0.833	0.750	0.829	0.236
	0.799	0.771	0.792	0.284
20 µg/mL	0.666	0.620	0.799	0.093
	0.646	0.754	0.768	0.134

**Table A2:** *p* value of each evaluated dose.

Concentration (µg/mL)	α	Probability Value *p*
20 µg/mL	0.05	0.00065535 *
10 µg/mL	0.05	0.00006810 *
2.5 µg/mL	0.05	0.00123425 *

* Statistically significant difference.

**Table A3:** Toxicity classification.

Doses	Chitosan	Blank	Formulation	Erlotinib
2.5 µg/mL	NC	LC	NC	LC
10 µg/mL	NC	LC	NC	SC
20 µg/mL	LC	LC	LC	SC

Where: NC: Non- cytotoxic. LC: Light cytotoxicity. MC: Moderate cytotoxicity. SC: Severe cytotoxicity.
